# Quantitative modeling of radioactive cesium concentrations in large omnivorous mammals after the Fukushima nuclear power plant accident

**DOI:** 10.1038/s41598-021-89449-0

**Published:** 2021-05-11

**Authors:** Igor Shuryak

**Affiliations:** Center for Radiological Research, Columbia University Irving Medical Center, 630 West 168th Street, VC-11-234/5, New York, NY 10032 USA

**Keywords:** Computational biology and bioinformatics, Ecology

## Abstract

Large quantities of radionuclides released by the Fukushima nuclear power plant accident entered terrestrial and marine ecosystems. The resulting radioactive contamination of large omnivorous wild mammals such as wild boar (*Sus scrofa*) and Asian black bear (*Ursus thibetanus*) varied greatly depending on location, season, and time after the accident. Quantitative modeling of how such factors influence radionuclide burdens in these species is important for enhancing current knowledge of chronic radionuclide exposure consequences in mammalian populations, and for assessing potential human risks from consumption of contaminated animal meat. Here we modeled the time course of radioactive cesium (^134^Cs + ^137^Cs) concentrations in boar and black bears from Fukushima Prefecture over ~ 7 years after the accident, using nonlinear robust and quantile regressions and mixed-effects modeling. To estimate predictive performance, models fitted to the full data set were compared with those fitted only to the first 3.5 years of data, and tested on the last 3.5 years of data. Ecological half-lives for radioactive cesium, and magnitudes and phase shifts for sinusoidal seasonal oscillations in cesium burdens, were estimated by each analysis method for each species. These results can improve the understanding and prediction of radionuclide concentrations in large mammals that inhabit radioactively contaminated areas.

## Introduction

Large-scale accidents that contaminated vast areas with radioactive materials unfortunately occurred throughout the history of nuclear power and nuclear weapons development, and the effects of this contamination on various organisms were studied for several decades^1–4^. The Fukushima Daiichi Nuclear Power Plant accident in Japan in March 2011 is the most recent major nuclear disaster. It released large quantities of radionuclides, some of which were incorporated into the bodies of plants and animals, including wild game species hunted by humans. Detailed data on radioactivity deposition across the land, and concentrations of important radionuclides like ^137^Cs in various animal species, were gathered and provided for public access following the Fukushima accident, starting shortly after the event and extending over the subsequent years. Such data provide important opportunities for researchers to study how radioactive fallout is distributed in ecosystems and various species within them, and to use this information to enhance the knowledge gathered from previous nuclear disasters.

The Fukushima accident, similarly to the Chernobyl accident of 1986, caused a large land area to be abandoned by humans, leading to “rewilding” trends where areas previously strictly controlled by human activities became available for colonization by wild species, including large mammals^5^. However, all biota on contaminated land experienced chronic exposure to various forms of ionizing radiation (e.g. γ, β and α) due to irradiation from external sources as well as internal exposure from incorporation of radionuclides into the body. This situation created interactions between positive and negative factors, where new habitat and resources that become available for wildlife due to reduction of human activities generated opportunities for population growth^5,6^, whereas detrimental effects of radiation (e.g. impaired reproduction, genomic instability, elevated mutation rates, carcinogenesis) could cause decreased abundance or extinction^7,8^. Additional complexity in this system could be caused by interactions between radiation and other stressful factors, such as adverse environmental conditions, inter- and intra-specific competition^9^.

Large wild mammals are important to study in the context of radioactive contamination for several reasons. They are taxonomically and physiologically similar to humans and, therefore, have similar radiosensitivity. These factors make studies of adverse effects of chronic irradiation on large mammals potentially relevant for human radiation protection. In addition, some large wild mammals are actively hunted by humans and consumption of their contaminated meat can result in human exposures to ionizing radiation from ingested radionuclides. This process can be enhanced by the ability of large mammals to cover long distances (tens of kilometers) in search of food, mates, or during dispersal of individuals to new territories, whereby animals with high radionuclide burdens can migrate out from contaminated areas to cleaner human-populated areas^10^.

The wild boar (*Sus scrofa*) is an important example of a large mammal that is useful to study in radioactively contaminated lands. This widely distributed omnivorous animal is native to the areas of Europe and Asia contaminated by both the Chernobyl and Fukushima accidents. In both regions it is an important game species targeted by human hunters and eaten by local residents. Abandonment of contaminated areas by humans allowed boar to exploit these areas and reach large population sizes there, probably due to their rapid reproduction rate and omnivorous diet. This pattern occurred after both the Chernobyl and Fukushima accidents^5,6,11–14^. In the Fukushima accident area boar became particularly prevalent^5^, likely because natural predators like wolves (*Canis lupus*) are present in Chernobyl, but are extinct in Japan. In Fukushima Prefecture after the accident, boar were hunted deliberately to reduce their numbers and limit the damage that they can cause to agriculture and potentially to human health^11^. Radionuclide concentrations were measured in many boar individuals killed by these hunting programs, which provided large amounts of data on radionuclide incorporation in this species.

Wild boar are known to strongly accumulate radioactive isotopes like ^137^Cs and to exhibit large variations in radionuclide burdens between individuals^13–16^. These tendencies probably result from the life style and behavior of the species. Boar are omnivorous and consume a wide variety of food sources, which change by season and location^16,17^. During the warm months of late spring to early autumn, boar feed mainly on vegetation. During colder months they switch to burrowing for food in the litter and soil, such as roots, tubers, mushrooms, and invertebrates. These foods extracted from soil tend to have higher radionuclide burdens than green plants, which explains why radioactive cesium levels in boar tissues tend to be higher in winter than in summer, both in Europe and in Japan^16,17^. Some mushrooms, such as deer truffles (genus *Elaphomyces*) are particularly strong bio-accumulators of radioactive materials, and consumption of these mushrooms by boar (which varies seasonally and between individuals) is believed to substantially contribute to radioactive cesium contamination in European boar after the Chernobyl accident^18^.

Boar can have large home ranges, which vary in size depending on food availability, environmental conditions, and human activities^11^. They mainly inhabit forest and forest edge habitats, but can travel long distances (many kilometers), for example in search of food such as agricultural fields^11^. Such mobility, in addition to variations in diet composition, are likely to contribute to the following phenomena: (1) very high variability in radionuclide burdens between wild boar individuals killed in the same area, (2) poor correlations between average soil radioactive contamination levels and radionuclide levels in tissues of boar from the same location^11,14^. In other words, radionuclide burdens in the bodies of boar from a given population in a contaminated area can vary by several orders of magnitude due to heterogeneous (“patchy”) soil contamination patterns, and to seasonal and inter-individual variability in radionuclide intake.

The Asian black bear (*Ursus thibetanus*) is another large omnivorous mammal species that inhabits the Fukushima accident area. This bear was formerly widely distributed in Asia, but is now under threat from hunting and habitat loss due to human activities. Asian black bears prefer forests, including mountainous terrain, and can have large home ranges. They exploited abandoned lands after the Fukushima accident, but most stayed further inland from the power plant, relative to wild boar.

Like the boar, the Asian black bear feeds on a wide variety of vegetation, acorns, nuts, and invertebrates, and occasionally attacks large mammals. To obtain these foods, the bear can forage at ground level, dig in the soil, and climb trees. Black bear feeding patterns change with the seasons. For example, consumption of seeds and nuts increases in the autumn, and this correlates with increased radioactive cesium burden in bear tissues^16^. Many black bear individuals killed by hunters in the Fukushima area were studied for radionuclide concentrations^5,16^.

The activity concentrations of radioactive cesium isotopes (^134^Cs and ^137^Cs) in wild boar and Asian black bears from the area affected by the Fukushima accident were studied by several authors^11,12,16,19,20^. The ranges of radioactive cesium levels and resulting radiation dose rates were very wide in both species. For example, Nemoto et al*.*^16^ estimated a mean dose rate of 0.103 μGy/h and a range of 0.007 to 0.477 μGy/h for black bears, and a mean of 0.479 μGy/h and a range of 0.010 to 12.700 μGy/h for wild boar.

These variations over several orders of magnitude, as well as seasonal oscillations, complicate the important task of quantitative modeling of time trends in radioactive cesium burdens in these large mammal species after the Fukushima accident. Here we analyzed the latest publicly available data for these species, which extend to approximately 7 years after the accident, to obtain a clearer understanding of the time course of radioactive cesium (^137^Cs and ^134^Cs) levels in boars and bears. We focused on quantifying the effects of ecological processes (e.g. migration of radionuclides from top soil into deeper layers, bioaccumulation of radionuclides in fungi and other food sources for large mammals, human land decontamination efforts, etc*.*) and seasonal oscillations in radionuclide uptake and excretion, which act in addition to physical decay of these radionuclides. We also applied the same modeling concepts to data on radioactive cesium contamination in wild boar from the Chernobyl accident area. Our estimates for the ecological half-lives and seasonal oscillation amplitudes of radioactive cesium concentrations in boar and bears, obtained using the techniques of robust regression, quantile regression, and mixed effects modeling, extend and complement the results of earlier studies.

## Materials and methods

### Data sets

Radioactivity measurement data for several species of wild game mammals and birds in Fukushima Prefecture from May 2011 to March 2018 were released to the public by the Fukushima Prefecture Government (https://emdb.jaea.go.jp/emdb/en/portals/1040501000/). We extracted the data for wild boar (*Sus scrofa*), 1404 samples, and Asian black bear (*Ursus thibetanus*), 422 samples. The resulting boar and bear data sets contained total radioactive cesium activity (^134^Cs + ^137^Cs isotopes) values (in Bq/kg) from animals captured at different times and locations within Fukushima Prefecture. The data were imported for analysis into *R* 4.0.3 software^21^.

We ln-transformed the cesium activity values to bring their distribution closer to normal, creating the variable LnCsTot. To facilitate regression analyses (described below), we removed instances of missing data and cesium levels below detection: 20 samples (1.4%) for boar and 15 samples (3.3%) for bears. The time when each sample was taken (labeled “Day of collection” in the Fukushima Prefecture Government data set) was converted to years since the Fukushima accident (since March 11, 2011), assuming that 1 year = 365.25 days. This time of sample collection in years was called variable *T*.

Since for each sample some time passed between sample collection and radioactivity measurement (labeled “Result found Date”, called *T*_*r*_ in our notation), we needed to correct the reported LnCsTot values for physical decay over this time, which was different for different samples. The procedure used to perform this correction is described in Supplementary methods. The data with corrected total cesium values (*LnCs*_*c*_) are provided in Supplementary data (Supplementary_Dataset_File_Full).

### Mathematical model

To describe the data on ln-transformed total radioactive cesium levels (*LnSc*_*c*_) in each species as function of time after the accident (*T*), we developed the following simple mathematical model (Eqs. 1A, 1B):

1A $${LnCs}_{c}=X+Q-\mu \times {T}^{\nu }+A\times \mathrm{sin}\left[2\times \pi \times \left(T+P\right)\right], $$

1B $$X=\mathrm{ln}\left[\mathrm{exp}\left(LnCs{134}_{t{0}_{r}}\right)\times {2}^{-\frac{T}{{Th}_{Cs134}}}+\mathrm{exp}\left(LnCs{137}_{t{0}_{r}}\right)\times {2}^{-\frac{T}{{Th}_{Cs137}}}\right]$$

Here the term *X* represents the estimated average radioactive cesium level in the studied area, based on the intercepts (*LnCs*134_*t0r*_ for ^134^Cs and *LnCs*137_*t0r*_ for ^137^Cs, respectively) from robust regression discussed in Supplementary methods, and taking into account only physical decay for each isotope (with half-lives of *Th*_*Cs*134_ for ^134^Cs and *Th*_*Cs*134_ for ^137^Cs, respectively). The terms *Q*, µ, ν, *A* and *P* represent adjustable model parameters. Parameter *Q* represents the fitted relationship between radioactive cesium levels in the animal (Bq/kg), relative to the external environment (Bq/m^2^). Parameter µ represents the net rate of radioactive cesium reduction in animal tissues over time due to all processes except physical decay (e.g. decrease in bioavailability due to migration of cesium into deeper soil layers, human-mediated cleanup efforts, *etc*.). Parameter ν is a potential power dependence for these processes. By default, ν was set to ν = 1, but exploratory calculations using ν = 2 or treating ν as a freely adjustable parameter (≥ 0.1) were performed as well. Parameters *A* and *P* in the sine function represent a sinusoidal approximation for seasonal changes in radioactive cesium levels in animal tissues (e.g. due to seasonal variations in diet and life style), where *A* is the amplitude of the oscillations, *P* is the phase shift, and the period is set to 1 year. For simplicity, these parameters were assumed to be the same for both studied cesium isotopes. The descriptions of each parameter are also presented in Table 1.

**Table 1:** The meanings of all parameters used in our mathematical model (Eq. 1A, 1B) for radioactive cesium levels in wild boar (*Sus scrofa*) and Asian black bear (*Ursus thibetanus*).

Model parameter	Units	Interpretation
Q	ln[m^2^/Bq]	Parameter *Q* represents the relationship between ln-transformed radioactive cesium levels in animal tissues, relative to the external environment
µ	Years^−1^	Parameter µ represents the net exponential rate of radioactive cesium reduction in animal tissues over time due to all processes except physical decay
ν	None	Parameter ν is a potential power dependence for these processes
A	ln[Bq/kg]	Parameter *A* is the amplitude of seasonal sinusoidal oscillations of ln-transformed radioactive cesium levels in animal tissues
P	Years^−1^	Parameter *P* is the phase shift of seasonal sinusoidal oscillations of ln-transformed radioactive cesium levels in animal tissues

### Model fitting approaches

Initially, we used nonlinear ordinary least squares (OLS) regression (*nls* R function) to fit the model (Eq. 1A, 1B) to the data. To find the global optimum fit, we repeated the fitting procedure 2000 times with slightly different random initial parameter values and recorded the solution with the smallest root mean squared error (RMSE). Diagnostics on this regression included checking of convergence criteria and analyses of residuals (by scatter plot and histogram, regressing residuals as function of *T*, visualizing the QQ plot, autocorrelation and partial autocorrelation functions with 95% confidence intervals, performing the Shapiro–Wilk normality test, and calculating skewness and kurtosis). For boar data, diagnostics revealed problems with convergence (both X-convergence and relative convergence) and non-normality of residuals: e.g. Shapiro–Wilk p-value = 1.476 × 10^–7^, skewness = − 0.37, kurtosis = 3.50. For black bear data similar problems occurred with convergence, but residuals were closer to the normal distribution (perhaps due to smaller sample size): e.g. Shapiro–Wilk p-value = 0.0526, skewness = − 0.058, kurtosis = 2.45.

Due to these issues, we used robust nonlinear regression (*nlrob R* package) to reduce the effects of “outlier” data points. To find the global optimum, we repeated the fitting procedure 2000 times with slightly different random initial parameter values and selected the solution with the smallest absolute value of median residuals. The best-fit parameters for OLS and robust regressions were somewhat different for both boar and bear data. For boar data, the minimum robustness weight was 0.339 and the median was 0.762, and the corresponding values for black bear data were 0.557 and 0.821, respectively.

For each species, we compared the performances of model variants with different assumptions about parameter ν: (1) The default case with ν = 1, which represents an exponential rate of radioactive cesium decrease due to processes other than physical decay. (2) The case with ν = 2, which represents quadratic decay. (3) The case with ν being freely adjustable (≥ 0.1). The comparisons were based on Akaike information criterion (AIC)^22,23^. The purpose of these calculations was to better assess the shape of the time course for non-physical factors involved in radioactive cesium level decline in animal tissues over time after the accident.

In addition to analyzing the full data set for each species, we also performed separate analyses on subsets of data from specific locations: from those districts of Fukushima Prefecture where the mean radioactive cesium levels in animal samples were the highest, and where a sufficiently large number of samples was present. For wild boar, the two selected districts for this subset analysis were Soso and Kenpoku (819 samples), and for black bear they were Kenpoku and Kenchu (163 samples).

To further assess the sensitivity of model results to geographical and temporal factors, we also constructed a separate subset of data for each species. This subset excluded data from the Aizu and Minamiaizu districts, which are separated by mountains from the Fukushima Daiichi Nuclear Power Plant, and excluded data collected ≤ 6 months after the accident. These restrictions were intended to determine model performance on data collected in a more geographically contiguous area after the initial abrupt changes in contamination levels were completed and the system entered the phase of more stable kinetics. The purpose of all these analyses was to assess whether the time course of radioactive cesium levels in the bodies of each species differed between locations with high contamination vs. those with lower contamination, and as function of time after the accident.

We were interested in quantifying not only the center of the distribution of radioactive cesium values in each species over time, but also in assessing the lower and upper tails of this distribution. For this purpose, we fitted the model (Eq. 1A, 1B) for each species using quantile regression (*nlrq* function in *quantreg R* package) for the median (50th percentile), and also for the 25th and 75th percentiles. Initial parameter estimates for the quantile regressions were taken from best-fit parameters from robust regression described above. The 25th and 75th percentiles were selected instead of more extreme values (e.g. 5th and 95th) because the latter resulted in poor quality fits due to limited amounts of data at the fringes of the distribution.

To assess the variability of model parameters by location in more detail, we used mixed effects modeling (*nlme R* package) on the data from each species. Since original OLS fits suggested substantial deviations of residuals from the normality assumption, we performed mixed effects modeling on data with some outlier data points removed. The *OutlierDetection* package in *R* removed 43 boar samples and 5 bear samples. These outliers are listed in the Supplementary_outlier_data_points file. The remaining samples were used for mixed effects model fitting, but model performance metrics like coefficient of determination (R^2^) and RMSE were assessed on the full data set (with outliers included) for each species.

Since the Fligner-Killeen test of homogeneity of variances by district generated low p-values for both species (4.6 × 10^–14^ for boar and 0.018 for black bear), we allowed modelled variances to differ by district (using the *weights* option in *nlme*). We investigated several random effects structures for some or all model parameters, with randomness by district only, or by district and municipality within district. Model diagnostics were the same as for fixed effects OLS modeling described above, and also included boxplots of model residuals by district. The mixed effects model variants with different random effects structures were compared using the *anova* function in *R*, and also by assessing convergence criteria, normality of residuals, skewness, and kurtosis. Consequently, preferred mixed effects model variants were selected for the full data as well as for the subset of two districts with high radioactive cesium levels, separately for each species.

### Model extrapolation from training to testing data

To investigate how the robust and quantile regression fits of our model could extrapolate beyond the time range that was used for model fitting, we split the data for each species into "training" (early times) and "testing" (later times) parts. The split was done based on time since the accident (*T* variable), so that approximately ½ of the samples were assigned to the training and testing sets, respectively. For wild boar data, the training set included times between 0.20 and 3.45 years after the accident, and the testing set included times between 3.45 and 7.03 years. For black bear data, the training set included times between 0.42 and 3.46 years after the accident, and the testing set included times between 3.46 and 6.87 years.

We also evaluated an alternative approach to splitting the data, where the split was done randomly instead of by time. In other words, any data point regardless of time had an equal probability of being assigned to either the training or the testing data set. Both the training and testing data subsets generated by this random split included the complete time range. This approach was implemented in context of the sensitivity analysis described above.

For each species, robust and quantile regressions were fitted to training data, and their predictions were calculated for testing data. For robust regression, RMSE was calculated on testing data for two scenarios: (1) for the model fitted to training data only, and (2) for the model fitted over the entire data range (training + testing). These RMSE values for conditions 1 and 2 were compared to assess the quality of model extrapolation. Extrapolation performance for robust and quantile regressions was also assessed visually by plotting the model predictions and data.

### Application of the model to wild boar data from the Chernobyl accident area

To compare the results of our analysis of wild boar contamination with radioactive cesium in the area affected by the Fukushima accident with data from another location, we also analyzed wild boar data from the Chernobyl accident area. These data were published by Gulakov^14^ and contain summaries of ^137^Cs contamination levels in the muscles of 188 boar collected between 1991 and 2008 (i.e*.* from 5 to 22 years after the 1986 accident). Sampling was carried out in three zones with different land contamination levels with ^137^Cs. This data set provides important information on radioactive cesium contamination in wild boar in the Chernobyl area. Unfortunately, ^137^Cs measurements in each sampled boar were not provided by Gulakov^14^, and only summary statistics are available for each zone and year after the accident (Tables 1–3 in reference^14^): number of animals, mean, minimum and maximum ^137^Cs levels.

We could not apply the full model (Eq. 1A, 1B) to these summary data which lacked seasonality information and ^134^Cs data. However, we were able to perform a weighted linear regression to quantify the ecological half-life of ^137^Cs in Chernobyl boar and the relationship between ^137^Cs levels in the animals (Bq/kg), relative to the external environment (Bq/m^2^). The data used for this analysis, derived from Gulakov^14^, are provided in Supplementary data (Supplementary_Dataset_File_Full). They contain the following variables. Zone = location of sample collection (Alienation, Permanent control or Periodic control). Time = time in years after the Chernobyl accident. LnMeanCs = ln-transformed mean ^137^Cs level in boar muscle (Bq/kg). LnMeanCs_c = LnMeanCs − X, where X is ln-transformed ^137^Cs land contamination (Bq/m^2^) in the given zone, corrected for physical decay of ^137^Cs. Weight = weighting of each data point used for regression. Weight = number of animals/(ln[maximum ^137^Cs level] − ln[minimum ^137^Cs level])^2^. These approximately inverse-variance weights were normalized by the overall mean across all data points, so that the mean weight across all data points was set to 1.

These data were analyzed by weighted linear regression in *R*, where LnMeanCs_c was allowed to depend on Time and Zone variables. Model variants containing all possible combinations and pairwise interactions between these predictor variables were fitted and their performances were compared using the Akaike information criterion with correction for small sample size (AICc). These calculations were performed using the *glmulti R* package. Multimodel inference (MMI) was performed on this collection of fitted model variants. It resulted in the calculation of model-averaged parameter estimates, 95% CIs and importance scores, corrected for model selection uncertainty. Of main interest here were the intercept parameter, which is analogous to parameter *Q* in the full model (Eq. 1A, 1B), and the Time parameter, which is analogous to parameter µ in the full model. The ecological half-life for ^137^Cs was calculated based on the Time parameter.

## Results

We analyzed the wild boar data with robust regression using variants of our model formalism (Eq. 1A, 1B) with different power dependences on time: with parameter ν = 1, ν = 2, or freely adjustable ν. These variants represent different assumptions about the shape of the time dependence for the reduction in radioactive cesium levels in boar tissues over time after the accident due to all processes other than physical decay. The variants with ν > 1 had better support by AIC on the full data set than the ν = 1 variant, by up to 41.6 AIC units for the freely adjustable ν variant (where best-fit ν was 2.750 with standard error (SE) of 0.796). However, the simplest variant with parameter ν = 1, which assumes that reduction in radioactive cesium levels in boar tissues over time after the accident due to all processes other than physical decay follows simple exponential kinetics, had the best performance in terms of extrapolating from training to testing portions of the data set (i.e. from early times after the accident to later times). The RMSE value on testing data for this model version fitted to the training data only, divided by the RMSE value on testing data for the model version fitted to all the data (training plus testing) was only 1.062. In comparison, much higher RMSE ratios (> 1.4), which indicate poor extrapolation performance, were found for model variants with ν = 2, or with freely adjustable ν.

**Figure 1:**
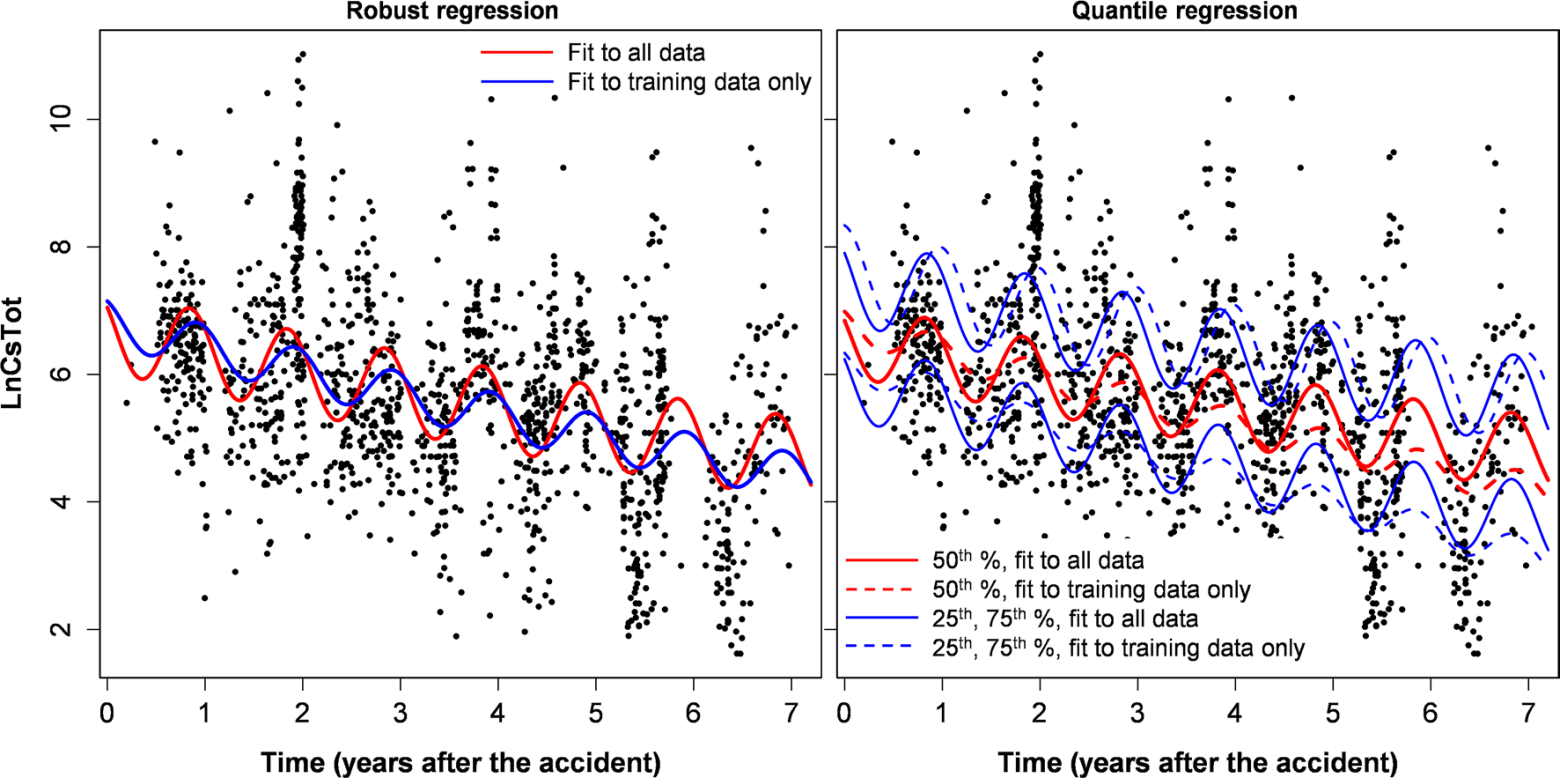
Robust and quantile regression analyses for wild boar (*Sus scrofa*) data. In this and the following figures, training data represent approximately ½ of the data set between 0 and ~ 3.5 years after the Fukushima accident, as described in the main text. The data points in both panels are the same, but some of them are obscured by the legend in the right panel. This figure was generated using *R* 4.0.3 software^21^.

**Figure 2:**
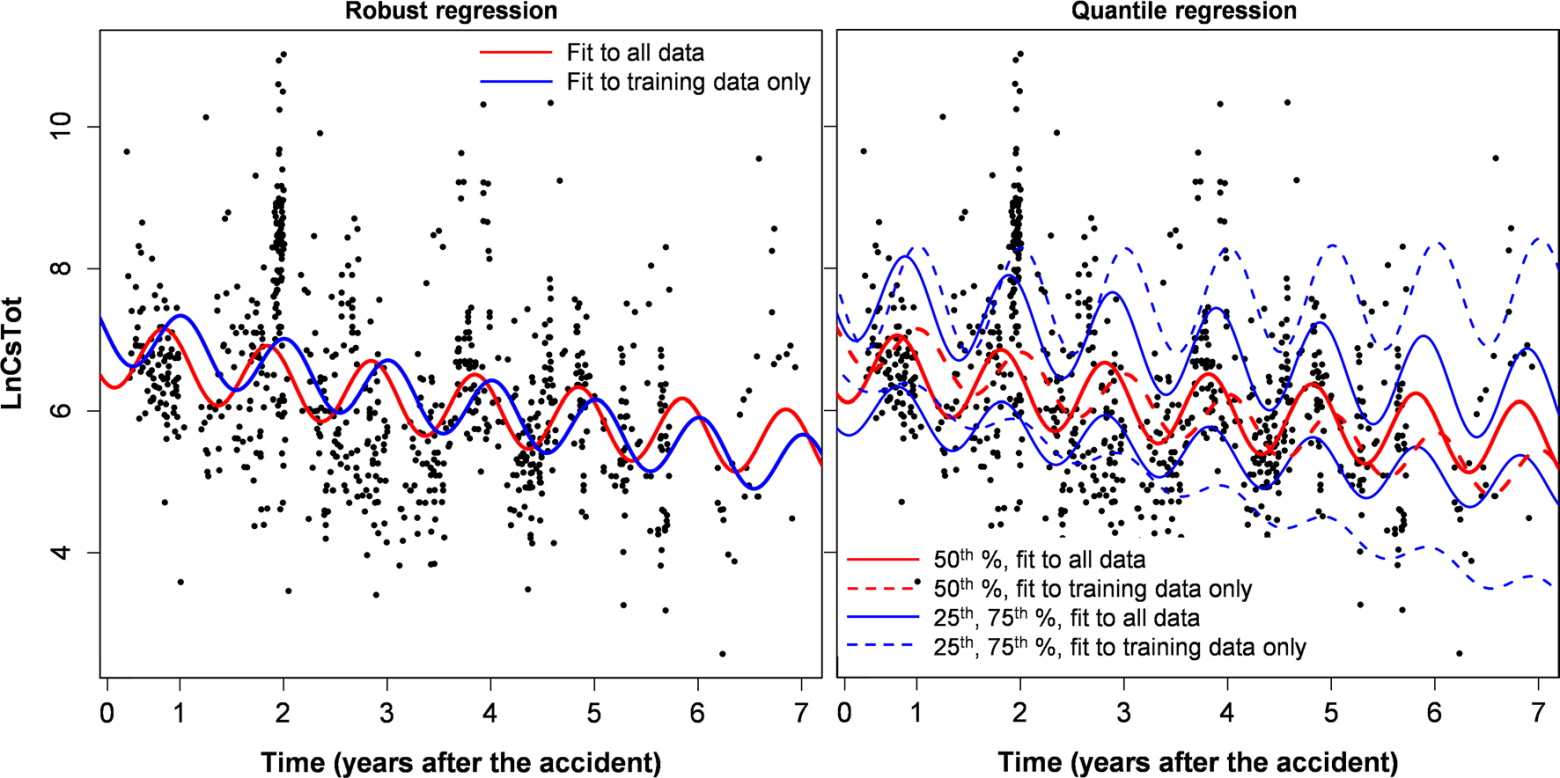
Robust and quantile regression analyses for wild boar (*Sus scrofa*) data for specific locations (Soso and Kenpoku districts) with high average radioactive cesium levels.

These results suggest that despite its worse AIC scores on the full data set, the simplest model version with ν = 1 had the best predictive power for extrapolating from short times to longer times. Due to this important property, which is visualized in the plots showing extrapolation of model predictions (Figs. 1, 2) and in the similarity of best-fit parameters for fits to the entire data set *vs*. fits to training data only (Tables 2, 3), and also to the model’s simplicity, we used this ν = 1 assumption for the quantile and mixed effects regression analyses of the boar data. The results of these analyses are shown in Figs. 1, 2 and in Tables 2, 3, 4.

**Table 2:** Parameter values for fitting our model (Eq. 1A, 1B) by robust regression to wild boar and Asian black bear data.

Fits to the full data set: parameter, best-fit value, SE			Fits to training data only: best-fit value, SE		Ratio of parameters for fits to training data only/full data: value, SE		Fits to the full data set for specific districts: best-fit value, SE		Fits to training data only for specific districts: best-fit value, SE		Ratio of parameters for fits to training data only/full data: best-fit value, SE	
**Boar**												
Q	− 4.851	0.097	−4.710	0.159	0.971	0.038	−4.646	0.110	−4.287	0.213	0.923	0.051
µ	0.173	0.025	0.234	0.071	1.349	0.453	0.085	0.031	0.179	0.104	2.091	1.428
A	0.641	0.059	0.357	0.077	0.556	0.131	0.477	0.064	0.440	0.106	0.924	0.255
P	0.405	0.017	0.325	0.044	0.802	0.112	0.395	0.025	0.225	0.046	0.570	0.122
**Black bear**												
Q	−6.012	0.170	−6.023	0.252	1.002	0.051	−5.222	0.222	−5.694	0.242	1.090	0.065
µ	0.130	0.036	0.144	0.098	1.105	0.807	0.214	0.044	0.051	0.092	0.240	0.431
A	0.568	0.141	0.373	0.185	0.657	0.365	0.390	0.191	0.278	0.164	0.712	0.546
P	0.267	0.032	0.353	0.075	1.323	0.325	0.276	0.063	0.542	0.124	1.964	0.634

In this and the following tables, the preferred model variant with parameter ν = 1 was used, and training data represent approximately ½ of the data set between 0 and ~ 3.5 years after the Fukushima accident, as described in the main text. SE represents standard errors. For wild boar, the two selected specific districts with high mean radioactive cesium levels were Soso and Kenpoku, and for black bear they were Kenpoku and Kenchu.

**Table 3:** Parameter values for fitting our model (Eq. 1A, 1B) by quantile regression to wild boar and Asian black bear data.

Per-cen-tile	Fits to the full data set: parameter, best-fit value, SE			Fits to training data only: best-fit value, SE		Ratio of parameters for fits to training data only/full data: value, SE		Fits to the full data set for specific districts: best-fit value, SE		Fits to training data only for specific districts: best-fit value, SE		Ratio of parameters for fits to training data only/full data: best-fit value, SE	
**Boar**													
50	Q	−4.894	0.089	−4.681	0.144	0.956	0.034	−4.751	0.102	−4.338	0.263	0.913	0.059
	µ	0.145	0.019	0.260	0.073	1.786	0.555	0.053	0.027	0.184	0.113	3.451	2.747
	A	0.582	0.060	0.262	0.076	0.451	0.138	0.526	0.061	0.378	0.210	0.719	0.407
	P	0.423	0.016	0.361	0.066	0.853	0.161	0.428	0.023	0.225	0.068	0.525	0.162
25	Q	−5.538	0.079	−5.281	0.127	0.954	0.027	−5.341	0.109	−4.762	0.146	0.892	0.033
	µ	0.209	0.021	0.320	0.055	1.534	0.303	0.058	0.031	0.353	0.074	6.124	3.502
	A	0.611	0.052	0.259	0.081	0.424	0.137	0.395	0.069	0.178	0.097	0.451	0.258
	P	0.415	0.017	0.399	0.057	0.962	0.144	0.420	0.026	0.264	0.109	0.628	0.262
75	Q	−3.972	0.152	−3.813	0.282	0.960	0.080	−3.706	0.169	−3.831	0.300	1.034	0.094
	µ	0.163	0.036	0.176	0.120	1.084	0.777	0.116	0.038	−0.115	0.171	−0.998	−1.517
	A	0.689	0.072	0.690	0.171	1.001	0.269	0.666	0.079	0.744	0.137	1.117	0.244
	P	0.398	0.025	0.267	0.041	0.671	0.112	0.353	0.022	0.239	0.045	0.676	0.134
**Black bear**													
50	Q	−6.065	0.187	-6.034	0.247	0.995	0.051	-5.010	0.221	−5.567	0.350	1.111	0.085
	µ	0.098	0.037	0.165	0.093	1.693	1.149	0.261	0.049	0.065	0.147	0.249	0.565
	A	0.663	0.252	0.248	0.195	0.374	0.326	0.323	0.252	0.298	0.226	0.923	1.004
	P	0.242	0.032	0.295	0.131	1.220	0.566	0.249	0.081	0.507	0.125	2.038	0.832
25	Q	−6.774	0.220	−6.618	0.268	0.977	0.051	−5.959	0.278	−6.094	0.307	1.023	0.070
	µ	0.107	0.064	0.112	0.112	1.043	1.218	0.189	0.054	0.029	0.108	0.156	0.573
	A	0.413	0.149	0.509	0.165	1.233	0.598	0.266	0.291	0.374	0.228	1.405	1.760
	P	0.282	0.065	0.325	0.043	1.151	0.305	0.230	0.131	0.554	0.106	2.409	1.444
75	Q	−5.201	0.230	−5.247	0.306	1.009	0.074	−4.567	0.328	−5.338	0.335	1.169	0.111
	µ	0.137	0.048	0.176	0.118	1.279	0.966	0.181	0.055	0.046	0.119	0.253	0.661
	A	0.577	0.153	0.208	0.244	0.360	0.433	0.544	0.260	0.408	0.264	0.750	0.604
	P	0.275	0.040	0.403	0.162	1.467	0.628	0.266	0.072	0.335	0.079	1.260	0.453

**Table 4:** Parameter values for fitting our model (Eq. 1A, 1B) by mixed-effects regression to wild boar and Asian black bear data.

Species	Para-meter	Fits to the full data set			Model performance metrics	Fits to the data for specific districts			Model performance metrics
		Fixed effects best-fit value	Fixed effects SE	Random effects SD		Fixed effects best-fit value	Fixed effects SE	Random effects SD	
Boar	Q	−5.574	0.491	0.584	R^2^ = 0.622	−4.147	0.683	0.820	R^2^ = 0.462
	µ	0.122	0.052	0.105	RMSE = 0.971	0.141	0.075	0.095	RMSE = 0.961
	A	0.472	0.100	0.256		0.336	0.048		
	P	0.420	0.013			0.422	0.025		
Black bear	Q	−5.646	0.477	0.339	R^2^ = 0.690	−5.749	0.403	0.423	R^2^ = 0.589
	µ	0.075	0.042	0.038	RMSE = 0.616	−0.023	0.084	0.183	RMSE = 0.640
	A	0.551	0.073			0.439	0.131		
	P	0.276	0.017			0.309	0.035		

The preferred mixed-effects model structure for each data set was selected based on several criteria described in the main text. Random effects for selected parameters were allowed to vary by district and municipality within district, and the variance was allowed to be different for each district. SE represents standard errors and SD represents standard deviations.

**Figure 3:**
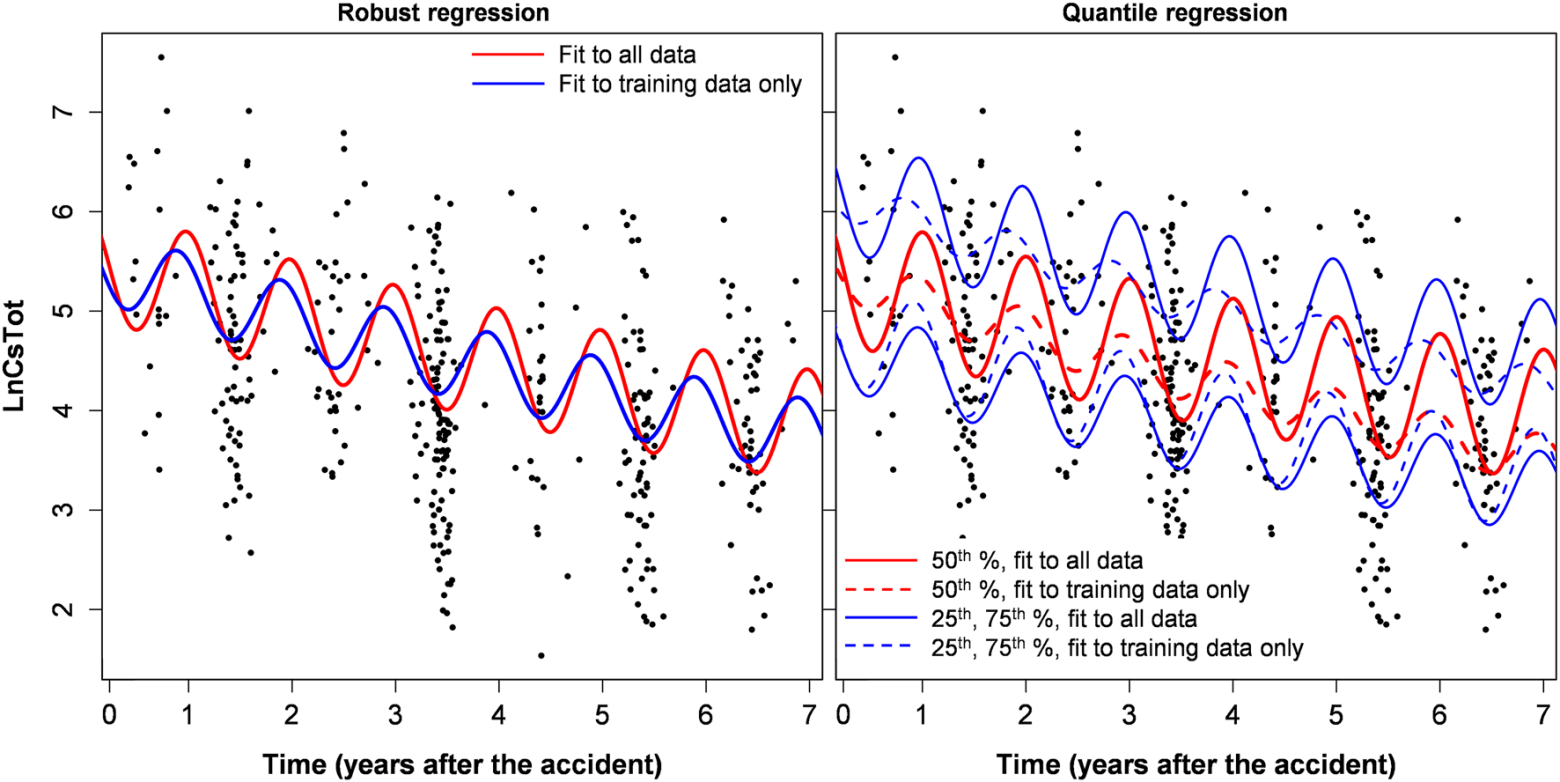
Robust and quantile regression analyses for Asian black bear (*Ursus thibetanus*) data. This figure was generated using *R* 4.0.3 software^21^.

**Figure 4:**
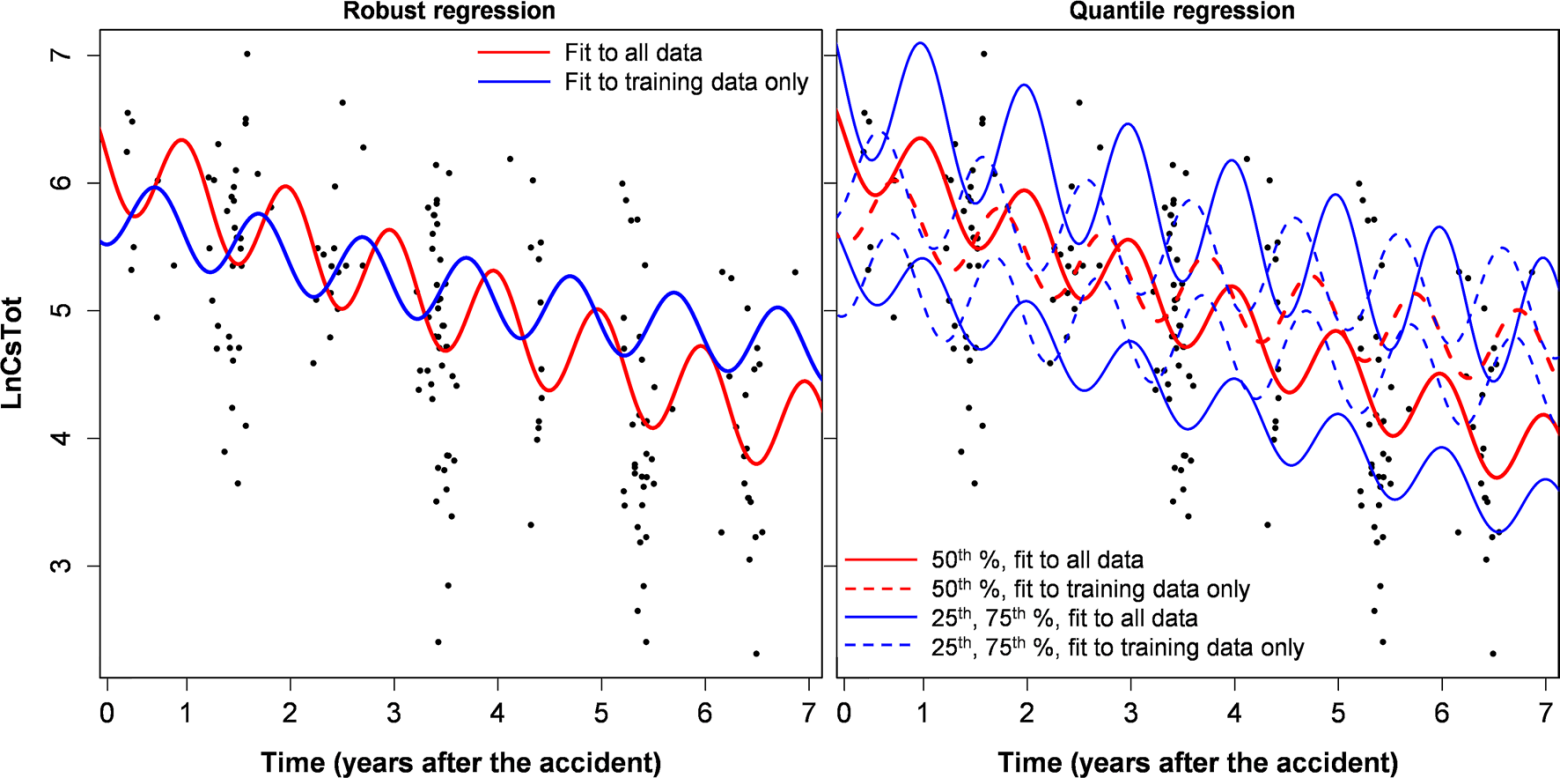
Robust and quantile regression analyses for Asian black bear (*Ursus thibetanus*) data for specific locations (Kenpoku and Kenchu districts) with high average radioactive cesium levels. This figure was generated using *R* 4.0.3 software^21^.

Model extrapolations from training to testing data of course resulted in some variations in estimated model parameters (Tables 2, 3 and Supplementary Tables 1–2). The variations were larger when the training/testing split was done based on time, compared with the sensitivity analysis, where the split was done randomly, as described in Materials and Methods. Although these parameter variations caused clearly visible effects when best-fit predictions were plotted graphically (Figs. 1, 2, 3, 4, Supplementary Figs. 1–2), they were generally not large, compared with corresponding parameter uncertainties (standard errors). This conclusion is supported by calculations of the ratio of parameters for fits to training data only / full data, which are shown in Tables 2, 3 and Supplementary Tables 1–2.

The best-fit parameters and visualization of model behaviors show that radioactive cesium levels in boar tissues continuously decreased over time after the accident. This decreasing trend could be reasonably extrapolated using our model from early to late times after the accident. Understandably, extrapolation quality was lower on subsets of the data for specific districts, especially for the tails of the distribution (25th and 75th percentiles). Similar results were obtained by our sensitivity analysis (described in Materials and Methods), where only a subset of data was used for each species (Supplementary Tables 1–2 and Supplementary Figs. 1–2). These findings suggest that the radioactive cesium burden in the boar population of Fukushima Prefecture is generally decreasing, but stable or increasing trends are potentially possible in some areas and/or for a fraction of individuals. Perhaps these patterns are caused by variations in bioavailability of cesium by location and/or different food source preferences between boar individuals.

Substantial seasonal oscillations in radioactive cesium levels in boar were evident, and they were reasonably modeled by the simple sinusoidal function (including this function in the model removed any significant autocorrelation of residuals). Peak radioactive cesium levels in boar tissues are predicted to occur in winter and early spring, probably due to increased reliance on food sources prone to cesium accumulation during these seasons. These findings are consistent with previous analyses^16^, but extend them to longer times after the accident.

Best-fit parameters for the robust, quantile and mixed effects analyses of boar data are compared in Table 5. For each method and subset of data, we calculated the ecological half-life of radioactive cesium in boar, defined as ln[2]/µ. As shown in Table 5, all regression methods produced similar ecological half-life values ranging from 3.3 to 5.7 years, but with considerable uncertainties. Analysis of the subset of data from specific districts (Soso and Kenpoku) with high radioactive cesium contamination produced somewhat higher estimates for the ecological half-life, from 4.9 to 13 years, but the uncertainties were expectedly larger due to reduced sample size, and included infinity in some cases (Table 5). The sensitivity analysis also produced similar ecological half-life estimates (Table 5). The seasonality effects (amplitude and phase shift) were very similar for all regression methods, for the full data set and for the subsets of locations (Table 5).

**Table 5:** Summary metrics for robust, quantile and mixed-effects fits of our model (Eq. 1A, 1B) to wild boar data.

Data set	Regression type	Ecological half-life (years)			Seasonality effect amplitude			Seasonality effect phase shift (months)		
		Best-fit value	95% CIs		Best-fit value	95% CIs		Best-fit value	95% CIs	
Full	Robust	4.000	3.128	5.548	1.898	1.693	2.129	4.863	4.469	5.256
	Quantile, 25th %	3.322	2.778	4.132	1.843	1.664	2.041	4.978	4.574	5.382
	Quantile, 50th %	4.768	3.803	6.388	1.790	1.590	2.015	5.072	4.691	5.454
	Quantile, 75th %	4.261	2.974	7.509	1.992	1.728	2.295	4.779	4.190	5.367
	Mixed effects	5.689	3.096	35.0	1.604	1.319	1.951	5.034	4.732	5.336
Specific districts	Robust	8.109	4.767	27.1	1.611	1.421	1.826	4.739	4.155	5.324
	Quantile, 25th %	12.02	5.887	∞	1.484	1.297	1.698	5.037	4.419	5.654
	Quantile, 50th %	13.00	6.509	5126	1.692	1.501	1.906	5.134	4.599	5.669
	Quantile, 75th %	6.001	3.654	16.78	1.947	1.668	2.272	4.232	3.722	4.742
	Mixed effects	4.917	2.400	∞	1.399	1.273	1.539	5.065	4.468	5.662
Sensitivity analysis	Robust	4.524	3.437	6.617	1.928	1.721	2.160	4.872	4.493	5.252
	Quantile, 25th %	3.656	2.875	5.018	1.823	1.620	2.051	4.959	4.490	5.429
	Quantile, 50th %	5.608	4.042	9.157	1.759	1.554	1.992	5.130	4.652	5.609
	Quantile, 75th %	5.080	3.347	10.53	2.012	1.729	2.342	4.738	4.131	5.345
	Mixed effects	3.934	2.197	18.84	1.579	1.252	1.993	5.118	4.808	5.427

In this and the following tables, infinite values for the ecological half-life of radioactive cesium indicate instances where the uncertainty on the corresponding model parameter µ extended to negative values. Sensitivity analysis represents analysis of a subset of data collected ≥ 6 months after the Fukushima accident, and where Aizu and Minamiaizu districts were excluded.

The parameter estimates shown in Table 5 suggest that radioactive cesium levels in wild boar in the Fukushima accident area vary by several orders of magnitude between boar individuals and are strongly influenced by seasonal effects. Importantly, the overall trends of decreasing radioactive cesium levels over time were detected by the implemented analyses and data selection approaches. The ecological half-life estimates and their uncertainties quantify these trends numerically. Of course, the inherent differences in assumptions and model fitting methodology between different regression methods lead to some variability in ecological half-life estimates. For example, robust regression estimated only the central tendency of the data, whereas quantile regression estimated three percentiles: 25th, 50th and 75th. In both robust and quantile regressions, the effects of geographical location were not explicitly modeled, and the entire area where data were collected was in essence treated as a single homogeneous unit. In contrast, mixed effects modeling accounted for the effects of location in more detail by allowing model parameter estimates to vary between different districts and municipalities within each district. However, compared with mixed effects modeling, robust and quantile regressions were less sensitive to “outlier” data points with extremely low or extremely high radioactive cesium levels. Each method therefore has its strengths and weaknesses, and model parameter estimates generated by each method should be compared and interpreted together to identify common tendencies.

The analogous analyses of black bear data also showed that the simplest model variant with parameter ν = 1 had the best extrapolation performance from training to testing data, with an RMSE ratio of only 1.04. The other variants had worse predictive power and worse AIC scores (by up to 2.7 units). The results of analyzing black bear data by robust, quantile and mixed effects regressions with the ν = 1 assumption are shown in Tables 2, 3, 4 and Figs. 3, 4. Best-fit parameters for all these models are compared in Table 6. These findings suggest that the ecological half-life values for radioactive cesium in black bears were not too different from those in wild boar, considering the large uncertainties (which were larger for the bear data due to lower sample size). The amplitudes of seasonal oscillations in cesium levels were also relatively similar for the two species, but the phase shifts tended to be different (Tables 5, 6). Details of mixed-effects model fits to wild boar and black bear data are provided in Supplementary material online (Supplementary_mixed_effects_model_output).

**Table 6:** Summary metrics for robust, quantile and mixed-effects fits of our model (Eq. 1A, 1B) to Asian black bear data.

Data set	Regres-sion type	Ecological half-life (years)			Seasonality effect amplitude			Seasonality effect phase shift (months)		
		Best-fit value	95% CIs		Best-fit value	95% CIs		Best-fit value	95% CIs	
Full	Robust	5.314	3.453	11.527	1.764	1.337	2.327	3.199	2.435	3.963
	Quantile, 25th %	6.471	2.977	∞	1.511	1.129	2.022	3.385	1.866	4.905
	Quantile, 50th %	7.105	4.056	28.60	1.941	1.183	3.183	2.903	2.159	3.646
	Quantile, 75th %	5.046	2.997	15.96	1.781	1.319	2.405	3.296	2.355	4.237
	Mixed effects	9.255	4.414	∞	1.735	1.504	2.001	3.316	2.910	3.722
Specific districts	Robust	3.237	2.312	5.398	1.478	1.016	2.149	3.313	1.832	4.795
	Quantile, 25th %	3.663	2.347	8.342	1.305	0.737	2.311	2.759	-0.313	5.831
	Quantile, 50th %	2.651	1.939	4.188	1.382	0.843	2.264	2.986	1.082	4.890
	Quantile, 75th %	3.833	2.402	9.481	1.723	1.035	2.868	3.192	1.496	4.888
	Mixed effects	∞	4.917	∞	1.551	1.201	2.003	3.713	2.893	4.534
Sensitivity analysis	Robust	3.161	2.212	5.533	1.651	1.124	2.427	3.769	2.652	4.885
	Quantile, 25th %	3.699	2.156	13.01	1.292	0.672	2.484	2.878	-0.081	5.837
	Quantile, 50th %	2.473	1.824	3.839	1.461	0.869	2.458	3.445	1.837	5.054
	Quantile, 75th %	4.039	2.488	10.73	1.792	1.178	2.724	3.648	2.016	5.281
	Mixed effects	–								

During sensitivity analysis, mixed effects models did not converge well due to limited data (only 163 samples), so no corresponding parameter values are shown.

Parameter *Q*, which is related to accumulation of radioactive cesium from the environment by each species, tended to be lower for black bears than for wild boar (Tables 2, 3). This finding is consistent with previous analyses^16^. The differences in best-fit *Q* values between the 75th and 25th quantiles were similar for both species: 1.565 for boar and 1.573 for black bears. These results suggest that the magnitude of variability of radioactive cesium burdens between individuals was similar in Fukushima boar and black bear populations.

We also applied our modeling concepts to ^137^Cs contamination data in wild boar from a completely different location: the Chernobyl accident area. As described in “Materials and methods”, these data were published by Gulakov^14^, and we analyzed them by weighted linear regression and multimodel inference (MMI). The best-supported weighted linear regression model variant, based on sample size corrected Akaike information criterion score, contained Time and Zone variables as predictors, without an interaction between them. The fitted coefficient for the Zone variable represents the relationship between mean ln-transformed radioactive cesium levels in boar tissues and in the external environment, which is analogous to parameter *Q* in our full model (Eq. 1A, 1B). Its best-fit values were: Alienation zone = − 5.552 ± 0.234 (standard error); Permanent control zone = 0.955 ± 0.186 units higher; and Periodic control zone = 1.438 ± 0.201 units higher. The fitted coefficient for the Time variable represents the time-dependent decrease in radioactive cesium level in boar tissues due to all processes except physical decay, which is analogous to parameter µ in our full model (Eq. 1A, 1B). Its best-fit value was 0.033 ± 0.020 years^−1^, which was not significantly different from zero (p-value = 0.115). The results of the MMI analysis, which included not only a single best-supported model, but averaged across all tested model variants, are shown in Supplementary Table 3 and in Supplementary Fig. 3.

These findings suggest that the relationship between mean ln-transformed radioactive cesium levels in boar tissues and in the external environment (*Q*) is similar in order of magnitude between Fukushima and Chernobyl data. However, the time-dependent decrease in radioactive cesium level in boar tissues (µ) in Chernobyl had 95% CIs that overlapped zero (Supplementary Table 3). The resulting ecological half-life estimate for ^137^Cs based on MMI analysis of Chernobyl data was 113.2 (95% CI: 18.5, ∞) years, and it was ∞ based on the single best-supported model variant mentioned above. This result suggests that ecological processes of radioactive cesium reduction in wild boar appear to be more rapid in Fukushima than in Chernobyl, although there is some overlap in the uncertainties of the estimates from these locations (e.g. the uncertainties of some ecological half-life estimates from Fukushima also included infinity, Table 5).

## Discussion

Our modeling analysis of wild boar and Asian black bear data from Fukushima Prefecture over approximately 7 years after the Fukushima Daiichi Nuclear Power Plant accident, using robust and quantile regressions and mixed-effects modeling techniques, generated detailed quantitative estimates for important parameters such as the ecological half-lives and seasonal oscillation magnitudes of radioactive cesium levels in each species (Tables 2, 3, 4, 5, 6). The mixed-effects models, which included the effects of geographical location, achieved relatively strong performance (R^2^ > 0.6 and RMSE < 1 Bq/kg on a natural log scale, Table 4), despite the simplicity of the underlying model structure (Eq. 1A, 1B) and only time and location as predictors.

Wild boars were quite abundant in the contaminated area, and > 1000 individuals killed by hunters provided detailed data on radioactive cesium burdens in this species at different times and locations. Our analysis estimated an ecological half-life for radioactive cesium in this species to be < 10 years, but with considerable uncertainties (Table 5). In contrast, in Europe some boar continued to have very high radioactive cesium concentrations in their tissues even several decades after the Chernobyl disaster, although the average levels of cesium isotopes in soil decreased dramatically over this time due to ecological processes and physical decay^14,15,24–26^. The finding of slow decline or even no decline in radioactive cesium levels due to ecological factors in European boar was also supported by our analysis of Chernobyl boar data (Supplementary Table 3 and Supplementary Fig. 3).

The persistence of high radioactive cesium levels in European boar populations despite their decline in soil was probably caused by increased availability of these radionuclides in boar diet over time. For example, some fungi (e.g. genus *Elaphomyces*) strongly accumulate radioactive cesium from soil and can serve as sources for boar contamination because these fungi are eaten by boar, particularly during colder months when green vegetation is not available. This process of boar contamination through consumption of fungi appears to be present, but considerably less pronounced in Japan, compared with Europe^16,19,24^. In addition, the level of radioactivity following the Fukushima accident decreased faster than in Chernobyl due to the effects of runoff and decontamination^27^.

Ecological half-lives of radioactive cesium in Fukushima Asian black bears were also estimated to be < 10 years, although uncertainties were larger than in boar because of smaller sample sizes (Table 6). A direct comparison with Europe is not possible for this species because it is found only in Asia, and has different ecology and diet than the European brown bear (*Ursus arctos*). Unlike wild boar, which feed actively throughout the year, Asian black bears in the Fukushima area hibernate in dens from November to April. Such periodic dormancy affects the seasonal oscillations in radioactive cesium levels in bear tissues, but our results (e.g. Figs. 1, 2, 3, 4) suggest that the sinusoidal pattern provides a decent description of these patterns. Qualitatively similar conclusions were reached in previous analyses of black bear data from Fukushima^16^.

The magnitudes and phases of seasonal oscillations in radioactive cesium levels in boar and black bears found in this study (Tables 5, 6) were similar to those reported by previous investigations^16^. Our estimates suggest that the seasonal oscillation amplitudes in these species are approximately 1.3 to 2.0 on the natural log scale (Tables 5, 6), which correspond to ~ 3.7 to 7.4-fold changes on the linear scale.

In conclusion, we developed a simple mathematical model of radioactive cesium (^137^Cs + ^134^Cs) levels in animal tissues (Eq. 1A, 1B) and fitted it to the most recent publicly available version of wild boar and Asian black bear data from Fukushima Prefecture, which covers approximately 7 years after the accident. We used several techniques to fit the model (robust regression, quantile regression and mixed effects modeling), which have different assumptions and provide complementary information. Specifically, model parameters estimated by robust regression are not very sensitive to “outlier” data points (i.e. extremely high or low radioactive cesium concentrations in some wild boar or black bear individuals). Parameters estimated by quantile regression are also relatively insensitive to outliers, and not only the central tendency, but several percentiles of the data distribution can be modeled. The strength of mixed effects modeling, as it was used here, is in its detailed treatment of parameter variations by geographical location, but its drawback is greater sensitivity to outliers than the previous methods. Importantly, ecological half-lives for radioactive cesium, and magnitudes and phase shifts for sinusoidal seasonal oscillations in cesium burdens, were estimated by each regression method for each species.

The study has limitations because many potentially important variables (e.g. age and sex of the animals, their home ranges and migration patterns) were not measured and, therefore, could not be included into the model. Aging and life span in wild boar were studied in European populations^28,29^, as well as in Japan^11,30^. In the Fukushima accident area, boars of all ages, from < 3 months to > 4 years, were found, and some studies reported a marginally statistically significant tendency for higher radioactive cesium levels in older individuals^11^. Unfortunately, information on age of the boar or black bears was not available in the data sets analyzed here, so age could not be incorporated as a predictor in the model.

Our approach was intended to describe the general tendencies in radioactive cesium contamination levels (e.g. median, 25th and 75th percentiles) over time in the studied species, but it cannot accurately predict the level in any specific individual. Despite these limitations, we believe that the study results can improve current understanding and prediction of radionuclide concentrations in large omnivorous mammals from radioactively contaminated areas.

## Supplementary Information

Supplementary Information.

Supplementary Dataset.

Supplementary Figure 1.

Supplementary Figure 2.

Supplementary Figure 3.

Supplementary Table 1.

Supplementary Information 1.

Supplementary Information 2.
