# Supplementary material for: Quantitative modeling of radioactive cesium concentrations in large omnivorous mammals after the Fukushima nuclear power plant accident
Source: Sci Rep. 2021 May 11;11:10049. doi: 10.1038/s41598-021-89449-0 (PMC8113437; doi:10.1038/s41598-021-89449-0)
Supplement: Supplementary file 1 — Supplementary Information. [file 41598_2021_89449_MOESM1_ESM.docx]

**Quantitative Modeling of Radioactive Cesium Concentrations in Large Omnivorous Mammals after the Fukushima Nuclear Power Plant Accident**

Igor Shuryak^1^

^1^Center for Radiological Research, Columbia University Irving Medical Center, New York, NY, USA

**Supplementary Methods**

To correct the reported LnCsTot values for physical decay over the time that passed between sample collection and radioactivity measurements (labeled “Result found Date”, called *T_r_* in our notation), we needed to estimate the contributions of ^134^Cs and ^137^Cs isotopes to the total measured radioactive cesium in each animal sample. For this purpose, we downloaded radioactive cesium deposition data for the studied area (from <https://emdb.jaea.go.jp/emdb/en/portals/b1020101/>) for 2012-2016, which provided separate measurements of ^134^Cs and ^137^Cs in units of Bq/m^2^. We ln-transformed these data and performed robust linear regression (using the *rlm* function in *R*) to estimate the time course for each isotope using a simple exponential model. For ln-transformed ^137^Cs the regression intercept (LnCs137_t0r_), which represents the fitted deposition of this isotope just after the accident, was 10.8190 with a standard error (SE) of 0.0484, and the regression slope (LnCs137_sr_), which represents the exponential rate of decrease in the level of this isotope over time in years after the accident due to both physical decay and other processes, was 0.0643 with SE of 0.0152. For ln-transformed ^134^Cs the regression intercept (LnCs134_t0r_) was 10.8769 with SE of 0.0482, and the regression slope (LnCs134_sr_) was 0.3884 with SE of 0.0152. In this notation the subscript *r* indicates robust regression. We also performed an analogous analysis of these data by quantile regression (using the *quantreg R* package) to estimate the time course for the median of each isotope. The resulting intercept and slope values (labeled with subscript *q* for quantile regression) for each isotope were similar to those from robust regression, but slightly lower. They were: LnCs137_t0q_ = 10.75064, SE = 0.06994, LnCs137_sq_ = 0.06843, SE = 0.01963; LnCs134_t0q_ = 10.79242, SE = 0.07114, LnCs134_sq_ = 0.38867, SE = 0.01964.

Based on these intercept and slope values, we estimated fractional contributions of the ^134^Cs isotope to the total radioactive cesium as function of time *T*. These calculations are shown below (Eqs. S1-S2), where the index *fr* represents the fractional contribution of ^134^Cs based on robust regression, and *fq* represents its fractional contribution based on quantile regression:

$\frac{{Cs134}_{f_{r}} = \exp\left[ {LnCs134}_{t0_{r}}-{LnCs134}_{s_{r}}\times T \right]}{\exp\left[ {LnCs134}_{t0_{r}}-{LnCs134}_{s_{r}}\times T \right]+\exp\left[ {LnCs137}_{t0_{r}}-{LnCs137}_{s_{r}}\times T \right]}$ (S1)

$\frac{{Cs134}_{f_{q}} = \exp\left[ {LnCs134}_{t0_{q}}-{LnCs134}_{s_{q}}\times T \right]}{\exp\left[ {LnCs134}_{t0_{q}}-{LnCs134}_{s_{q}}\times T \right]+\exp\left[ {LnCs137}_{t0_{q}}-{LnCs137}_{s_{q}}\times T \right]}$ (S2)

The corresponding fractional contributions of the ^137^Cs isotope, calculated by each method, were defined as 1 minus the ^134^Cs isotope contributions.

We calculated the geometric mean of the ^134^Cs fractional contributions estimated by robust and quantile regression methods as follows (Eq. S3):

${Cs134}_{fg} =\sqrt{{Cs134}_{f_{r}}{\times Cs134}_{f_{q}}}$ (S3)

This calculation was then used to correct the reported total cesium measurements in animal tissues for physical decay from the time of reading (*T_r_*) back to the time of collection (*T*). The corrected values for each isotope (*Cs*134*c* and *Cs*137*c*) and for total radioactive cesium (*LnSc_c_*) are described below (Eqs. 3A-3C), where *Th* indicates the physical decay half-life of each isotope (2.0652 years for ^134^Cs and 30.1671 years for ^137^Cs):

$Cs{134}_{c}=\exp\left[ LnCsTot \right]\times{Cs134}_{fg}\times2^{\left( \frac{\left[ T_{r}-T \right]}{{Th}_{Cs134}} \right)}$ (S4)

$Cs{137}_{c}=exp[LnCsTot]\times{(1-Cs134}_{fg})\times2^{([T_{r}-T]/{Th}_{Cs137})}$ (S5)

${LnCs}_{c}=ln[Cs{134}_{c}+Cs{137}_{c}]$ (S6)
