# Supplementary material for: Quantitative modeling of radioactive cesium concentrations in large omnivorous mammals after the Fukushima nuclear power plant accident
Source: Sci Rep. 2021 May 11;11:10049. doi: 10.1038/s41598-021-89449-0 (PMC8113437; doi:10.1038/s41598-021-89449-0)
Supplement: Supplementary file 6 — Supplementary Table 1. [file 41598_2021_89449_MOESM6_ESM.docx]

**Quantitative Modeling of Radioactive Cesium Concentrations in Large Omnivorous Mammals after the Fukushima Nuclear Power Plant Accident**

Igor Shuryak^1*^

^1^Center for Radiological Research, Columbia University Irving Medical Center, New York, NY, USA

^*^ Corresponding author: Igor Shuryak, M.D., Ph.D.

Center for Radiological Research, Columbia University,

630 West 168^th^ street, VC-11-234/5, New York, NY, 10032

Phone: 212-305-2405; Fax: 212-305-3229

E-mail: [is144@cumc.columbia.edu](mailto:is144@cumc.columbia.edu)

**Supplementary Figure 1. Robust and quantile regression analyses of the subsets of wild boar (*Sus scrofa*) data selected for sensitivity analyses.** The procedure for selecting this data subset is described in the main text. In this and the following figures, training data represent, as described in the main text. The data points in both panels are the same, but some of them are obscured by the legend in the right panel. This figure was generated using *R* 4.0.3 software ^21^.

**Supplementary Figure 2. Robust and quantile regression analyses of the subsets of Asian black bear (*Ursus thibetanus*) data selected for sensitivity analyses.** This figure was generated using *R* 4.0.3 software ^21^.

**Supplementary Figure 3. Multimodel inference results using weighted linear regression analysis of ^137^Cs contamination levels in wild boar from the Chernobyl accident area.** These data and analysis methods are described in the Materials and Methods section of the main text. The LnMeanCs_c variable represents the ln-transformed contamination level, relative to the external environment. Red symbols = data from the Alienation zone. Blue symbols = data from the Permanent control zone. Green symbols = data from the Periodic control zone. Black curve = regression fit. This figure was generated using *R* 4.0.3 software ^21^.

**Supplementary Table 1. Parameter values for fitting our model by robust regression to subsets of wild boar and Asian black bear data selected for sensitivity analyses.** The procedure for selecting these data subsets is described in the main text. SE = standard error.

| **Species** | **Fits to the full data set** | | | **Fits to training data only** | | **Ratio of parameters for fits to training data only / full data** | | **RMSE ratio on testing data: model fitted to training data only / model fitted to full data** |
| --- | --- | --- | --- | --- | --- | --- | --- | --- |
|  | **Para-meter** | **Best-fit value** | **SE** | **Best-fit value** | **SE** | **Best-fit value** | **SE** |  |
| **Boar** | **Q** | -4.844 | 0.096 | -4.880 | 0.137 | 1.008 | 0.035 | 1.004 |
|  | **µ** | 0.153 | 0.025 | 0.146 | 0.035 | 0.953 | 0.277 |  |
|  | **A** | 0.657 | 0.058 | 0.580 | 0.082 | 0.883 | 0.147 |  |
|  | **P** | 0.406 | 0.016 | 0.408 | 0.027 | 1.005 | 0.077 |  |
| **Black bear** | **Q** | -5.079 | 0.221 | -5.011 | 0.308 | 0.987 | 0.074 | 1.024 |
|  | **µ** | 0.219 | 0.048 | 0.255 | 0.067 | 1.162 | 0.397 |  |
|  | **A** | 0.502 | 0.196 | 0.485 | 0.260 | 0.966 | 0.641 |  |
|  | **P** | 0.314 | 0.047 | 0.348 | 0.067 | 1.108 | 0.271 |  |

**Supplementary Table 2. Parameter values for fitting our model by quantile regression to subsets of wild boar and Asian black bear data selected for sensitivity analyses.** The procedure for selecting these data subsets is described in the main text. SE = standard error.

| **Species** | **Per-cen-tile** | **Fits to the full data set** | | | **Fits to training data only** | | **Ratio of parameters for fits to training data only / full data** | |
| --- | --- | --- | --- | --- | --- | --- | --- | --- |
|  |  | **Para-meter** | **Best-fit value** | **SE** | **Best-fit value** | **SE** | **Best-fit value** | **SE** |
| **Boar** | 50 | **Q** | -4.902 | 0.099 | -4.863 | 0.126 | 0.992 | 0.033 |
|  |  | **µ** | 0.124 | 0.024 | 0.122 | 0.032 | 0.990 | 0.326 |
|  |  | **A** | 0.565 | 0.063 | 0.470 | 0.069 | 0.832 | 0.154 |
|  |  | **P** | 0.428 | 0.020 | 0.421 | 0.029 | 0.984 | 0.083 |
|  | 25 | **Q** | -5.520 | 0.099 | -5.529 | 0.143 | 1.002 | 0.031 |
|  |  | **µ** | 0.190 | 0.026 | 0.189 | 0.035 | 0.999 | 0.232 |
|  |  | **A** | 0.600 | 0.060 | 0.538 | 0.085 | 0.896 | 0.168 |
|  |  | **P** | 0.413 | 0.020 | 0.419 | 0.033 | 1.015 | 0.093 |
|  | 75 | **Q** | -3.988 | 0.153 | -4.077 | 0.228 | 1.022 | 0.069 |
|  |  | **µ** | 0.136 | 0.036 | 0.145 | 0.051 | 1.064 | 0.465 |
|  |  | **A** | 0.699 | 0.077 | 0.644 | 0.088 | 0.921 | 0.162 |
|  |  | **P** | 0.395 | 0.026 | 0.445 | 0.037 | 1.127 | 0.119 |
| **Black bear** | 50 | **Q** | -4.885 | 0.237 | -5.080 | 0.322 | 1.040 | 0.083 |
|  |  | **µ** | 0.280 | 0.051 | 0.270 | 0.061 | 0.964 | 0.279 |
|  |  | **A** | 0.379 | 0.265 | 0.233 | 0.341 | 0.613 | 0.997 |
|  |  | **P** | 0.287 | 0.068 | 0.363 | 0.177 | 1.263 | 0.686 |
|  | 25 | **Q** | -5.966 | 0.308 | -5.499 | 0.340 | 0.922 | 0.074 |
|  |  | **µ** | 0.187 | 0.068 | 0.238 | 0.078 | 1.268 | 0.624 |
|  |  | **A** | 0.256 | 0.334 | 0.659 | 0.424 | 2.573 | 3.742 |
|  |  | **P** | 0.240 | 0.126 | 0.353 | 0.056 | 1.470 | 0.805 |
|  | 75 | **Q** | -4.509 | 0.279 | -4.441 | 0.535 | 0.985 | 0.133 |
|  |  | **µ** | 0.172 | 0.055 | 0.236 | 0.105 | 1.374 | 0.751 |
|  |  | **A** | 0.583 | 0.214 | 0.655 | 0.334 | 1.124 | 0.706 |
|  |  | **P** | 0.304 | 0.069 | 0.373 | 0.099 | 1.227 | 0.430 |

**Supplementary Table 3. Parameter values for multimodel inference results on weighted linear regression fits to ^137^Cs contamination level data from wild boar in the Chernobyl accident area.** The intercept parameter is analogous to parameter *Q* in the full model (Eq. 1 in the main text), and the Time parameter is analogous to parameter µ in the full model. The data used for this analysis are provided in Supplementary data (Supplementary_Dataset_File_Full).

| **Model parameter** | **Best-fit value** | **95% CIs** | | **Importance score** |
| --- | --- | --- | --- | --- |
| Intercept (Q) | -4.117 | -4.764 | -3.470 | 1.000 |
| Time (µ) | -0.006 | -0.037 | 0.025 | 0.308 |
| ZonePermanent_control | 0.037 | -0.310 | 0.385 | 0.258 |
| ZonePeriodic_control | 0.147 | -0.395 | 0.689 | 0.258 |
| Time×ZonePermanent_control | 0.007 | -0.028 | 0.043 | 0.174 |
| Time×ZonePeriodic_control | 0.008 | -0.026 | 0.043 | 0.174 |
| Time×ZoneAlienation | -0.002 | -0.012 | 0.008 | 0.087 |
